# Quantifying Short-Term Urban Land Cover Change with Time Series Landsat Data: A Comparison of Four Different Cities

**DOI:** 10.3390/s18124319

**Published:** 2018-12-07

**Authors:** Hongsheng Zhang, Ting Wang, Yuhan Zhang, Yiru Dai, Jiangjie Jia, Chang Yu, Gang Li, Yinyi Lin, Hui Lin, Yang Cao

**Affiliations:** 1 Institute of Space and Earth Information Science, The Chinese University of Hong Kong, New Territories, Hong Kong 999077, China; wangtingwhu@126.com (T.W.); yuhan_zhang_sky@yahoo.com (Y.Z.); daiyiru2014@163.com (Y.D.); Cavalryj@163.com (J.J.); godricyu@hotmail.com (C.Y.); ligang@cuhk.edu.hk (G.L.); yinyilin@link.cuhk.edu.hk (Y.L.); huilin@cuhk.edu.hk (H.L.); 2 Shenzhen Research Institute, The Chinese University of Hong Kong, Shenzhen 518000, China; 3 Hubei Geomatics Information Center, Hubei Bureau of Surveying, Mapping and Geoinformation, Wuhan 430000, China; 4 Jiangsu Academy of Science and Technology for Development, Nanjing 210042, China; 5 School of Computer Science, South China Normal University, Guangzhou 510631, China; yangcao@scnu.edu.cn

**Keywords:** seasonal, urban land cover, remote sensing, Landsat, impervious surface

## Abstract

Short-term characteristics of urban land cover change have been observed and reported from satellite images, although urban landscapes are mainly influenced by anthropogenic factors. These short-term changes in urban areas are caused by rapid urbanization, seasonal climate changes, and phenological ecological changes. Quantifying and understanding these short-term characteristics of changes in various land cover types is important for numerous urban studies, such as urbanization assessments and management. Many previous studies mainly investigated one study area with insufficient datasets. To more reliably and confidently investigate temporal variation patterns, this study employed Fourier series to quantify the seasonal changes in different urban land cover types using all available Landsat images over four different cities, Melbourne, Sao Paulo, Hamburg, and Chicago, within a five-year period (2011–2015). The overall accuracy was greater than 86% and the kappa coefficient was greater than 0.80. The R-squared value was greater than 0.80 and the root mean square error was less than 7.2% for each city. The results indicated that (1) the changing periods for water classes were generally from half a year to one and a half years in different areas; and, (2) urban impervious surfaces changed over periods of approximately 700 days in Melbourne, Sao Paulo, and Hamburg, and a period of approximately 215 days in Chicago, which was actually caused by the unavoidable misclassification from confusions between various land cover types using satellite data. Finally, the uncertainties of these quantification results were analyzed and discussed. These short-term characteristics provided important information for the monitoring and assessment of urban areas using satellite remote sensing technology.

## 1. Introduction

The monitoring and assessment of urban land cover changes are significant issues in global studies on topics, such as global warming and climate change [1,2,3]. Remote sensing has been playing an increasingly important role in the monitoring of global urban land cover changes [2,4,5,6]. With the increasing availability of free of charge satellite data, it is essential to use time series satellite remote sensing images to obtain land cover information to monitor and understand the dynamics. Previously, many studies have been carried out with multitemporal satellite images for the monitoring of various land use or land cover types, such as forest [7], water resources [8], wetlands [9,10,11], paddy rice, or other croplands [12,13]. Numerous methods or tools have been proposed for the detection and analysis of these land use or land cover changes, including cellular automata models [14], relative density ratio-based approach [15], Bradley-Terry model [16], unsupervised change detection method [17], principal component analysis [18], as well as the recent cloud computing platform Google Earth Engine [9,12,13].

In urban areas, many researchers have studied urban land cover changes over long time series to monitor the variations of different urban land cover types, evaluate the performance of some policies or measures taken by governments, or provide advice to government managers [19,20,21]. Quantitative analysis of time series satellite images at medium and coarse resolutions have been conducted with statistical methods and uncertainty modeling for land use cover changes [14,15,22]. The urban expansion of Beijing was analyzed from 1984 to 2013 at an annual frequency [19]. The land classification was based on a total of 123 Landsat images along with the Normalized Difference Vegetation Index (NDVI) time series data derived from these images. According to the results, the average growth rates of the urban region in Beijing for each decade over 30 years were 47.51 ± 4.17 km^2^/year, 34.65 ± 2.90 km^2^/year, and 99.48 ± 1.3 km^2^/year. Landsat Thematic Mapper (TM) images of the Pearl River Delta region with brightness, greenness, and wetness values were combined to obtain the changes in urban land cover classes between 1988 and 1996 [21]. The main result of the conversions from cultivated land to urban regions demonstrated the urban expansion and development of cities in the Pearl River Delta region. The variation of natural wetlands in Huixian, China was investigated based on a series of TM data in 1987, 1991, 2006, and 2009 through land cover classification [20]. The results showed that from 1987 to 2009, approximately half of the natural wetlands had been transformed into farmlands, and more than one-third of natural wetlands had been turned into artificial wetlands, which demonstrated the degradation of and damage to natural wetlands. From 2005 to 2009, the area of wetlands increased by 0.5 km^2^, which proved the adequate performance of the wetland restoration measures that were carried out by the local government. The dynamic changes in land cover and land use in the Manica Province in Mozambique were analyzed between 1990 and 2004 [23], and the results revealed that considerable amounts of miombo areas had been converted to cultivated lands during these 14 years, which indicated damage to natural forests. Additionally, croplands near the road networks with shifting cultivation types changed to more permanent types. Thus, the authors proposed that protecting natural ecosystems should be given more attention by the local government and society. In addition, time series satellite data were employed to analyze the temporal changes of urban areas in other cities, such as western Washington [24], Columbia [25,26], and Maryland [26,27] in the USA.

However, most previous studies focused on the long-term changes of land covers, while only a few studies looked at the short-term land cover changes [28,29,30,31,32,33,34]. In this study, long-term changes refer to the land cover changes in annual or longer time scale, while short-term changes refer to land cover changes within one year. Long-term land cover changes have been studied intensively using satellite images with time scales ranging from annual to decadal. It was recognized that land use or land cover changes not rapidly in the urban areas, as urbanization takes time to modify the land surface, and thus it is enough to evaluate the urban land use or land cover changes for every several years (e.g., five or ten years) [35,36,37,38]. Consequently, short-term changes in urban land covers were generally ignored in existing literature. However, short-term land cover changes occur in urban areas due to several factors: (1) fast spreading buildings and pavement in urban areas within one year or several months in rapid urbanized cities; (2) urban greening areas that have been increased by many governments to improve the urban environment; (3) bare lands generated during the construction procedure of buildings and pavement infrastructures in rapid urbanized cities; and, (4) variable source areas affected by the seasonal precipitation depending on climate conditions in different cities. All of these factors make the short-term changes possible in urban land covers, and these short-term changes can become important factors for the monitoring of urban areas using remote sensing technologies. For instance, in the application of urban sprawl assessments in every year or every several years, which seasons would be the best to choose satellite images since there may be many images along the whole year? To answer this question, the characteristics of short-term changes in different urban land covers should be understood first. Unfortunately, understanding the short-term changes of urban land covers is challenging, as it is often difficult to get sufficient data sets fulfilling the following requirements: (1) satellite images with high temporal resolution (e.g., monthly or seasonal) are needed; (2) long period of such high temporal resolutions observations are needed to increase the reliability of assessment results since only one year observations are not significant; and, (3) observations with different landscape patterns that may be an factor to the short-term changes. To overcome these challenges and explore the short-term changes in urban land covers, this study aims to more reliably investigate short-term variation patterns using all available time series Landsat images within five years in four selected cities and quantify the short-term land cover changes with Fourier series and the Sum of Sine Functions to fit the short-term changes.

## 2. Study Area and Datasets

### 2.1. Study Sites

**Figure 1:**
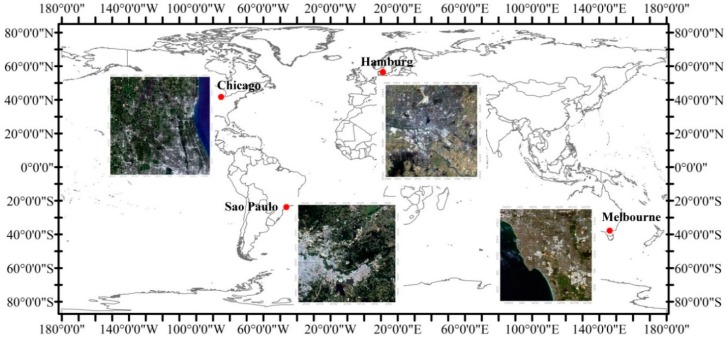
Geographic locations of the four selected cities, the images are true color composite of Landsat 8 data (R: band 4, G: band 3, B: band 2).

There are many cities with different natural and cultural characteristics that can be chosen as case studies for assessing short-term land cover changes. A number of climatic zones should also be considered as a selection criterion. However, to address short-term land cover changes, intensive available time series satellite data are first needed. Consequently, many cities with limited amounts of acceptable satellite data cannot be selected. Second, different natural conditions should be considered, especially the climatic zones that extensively contribute to the short-term land cover changes, such as seasonal vegetation changes. Third, culture can also be a factor of land cover changes, and thus, developed (Melbourne, Hamburg, and Chicago) and developing (Sao Paulo) cities were also considered. Last but not least, computational load was also an important factor, as the number of cities and number of images in one city should be balanced when studying short-term land cover changes. Finally, four typical urban areas from four different climate zones were selected, including Melbourne, Sao Paulo, Hamburg, and Chicago, and time-series satellite datasets over five years were used. According to the Köppen climate classification [39], which is one of the most popular climate classification systems, Melbourne experiences a temperate oceanic climate, Sao Paulo has a humid subtropical climate, Hamburg has a maritime temperate climate, and Chicago has a humid continental climate. With a comprehensive survey of available time-series satellite data, more available satellite datasets were provided in these four cities. Moreover, these cities presented a good combination regarding the selection criteria described above. Furthermore, when considering both high frequency and cloud-free datasets, areas of approximately 1500 km^2^ were selected in each of the four cities. This area was able to cover both the urban and suburban regions in the selected cities, and thus it includes all of the local land cover classes to be evaluated for short-term changes (Figure 1). The geographic locations of the four cities are shown in Figure 1.

**Figure 2:**
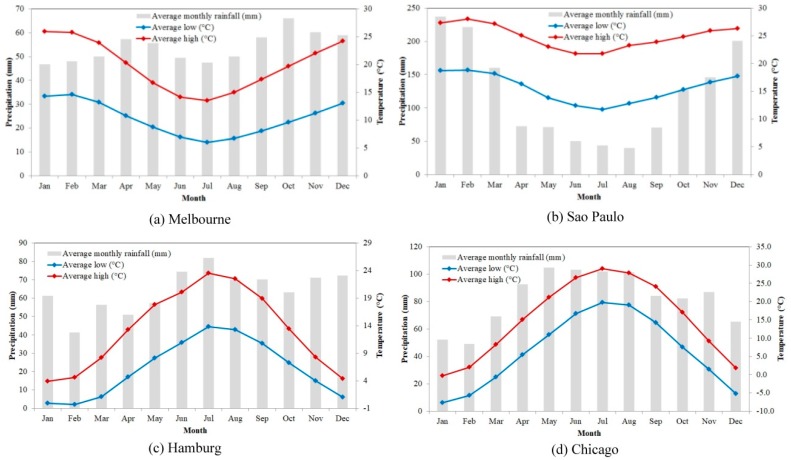
Climatic characteristics of the four selected cities (Data source: World Meteorological Organisation).

The climate characteristics of mean precipitation, mean maximal, and mean minimal temperature for each month are also illustrated in Figure 2. A brief description of the climate characteristics and urban development of each city is provided, as follows. Melbourne is the capital city of the Australian state of Victoria and the second most populous city in Australia [40]. Melbourne is located on the large natural bay of Port Phillip and expands into the hinterlands. Spring in Melbourne lasts from September to November, followed by three other seasons that are each three months long. Figure 2a shows that the temperature in Melbourne is relatively higher and the rainfall is relatively lower in the summer. Sao Paulo is the capital of Sao Paulo State in Brazil and it is populous with rapid urbanization, poor quality housing, and poor access to infrastructures, such as water, electricity, and sewage. Sao Paulo has a monsoon-influenced humid subtropical climate and four seasons that are not distinct. Generally, summer is from October to March and winter is from May to August (Figure 2b). Hamburg is one of the largest cities in Germany and the European Union. Hamburg is located in the center of Europe and it has become the center of German news media and industrial manufacturing. Hamburg is known as the gateway to Germany [41]. Hamburg is mild and humid all year. The annual rainfall is 774 mm and rainy days account for 52 days each year on average. In the fall and winter seasons, there is often stormy weather. The hottest month is July with an average temperature of 17.4 °C, and January is the coldest month with a monthly average temperature of 1.3 °C (Figure 2c). Chicago is a metropolitan city in Cook County, Illinois, USA. Under a humid continental climate, the characteristics of the four seasons are distinct. In Chicago, spring is mild, and it is also the wettest season, which spans from March to May. Deciduous vegetation begins to grow leaves in mid-April. Summer is hot and humid. Summer spans June, July, and August. The highest temperature of the year always appears in July. The low mean temperature of below freezing leads to snow coverage during most of the winter period (Figure 2d).

### 2.2. Satellite Data and Preprocessing

**Figure 3:**
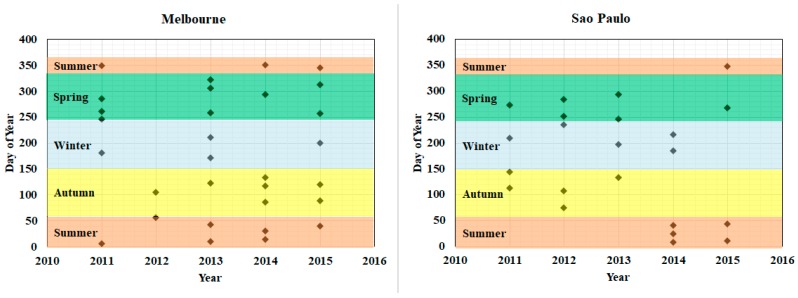

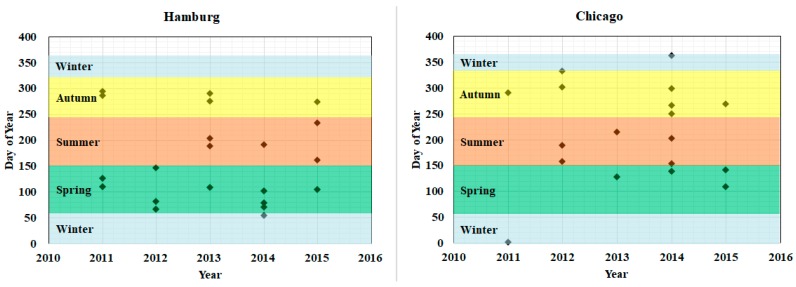
Temporal distribution of the imagery dates of selected Landsat images in the four cities, with each dot denoting one image and each color representing one season.

The data sources in this study included Landsat Thematic Mapper (TM), Landsat Enhanced Thematic Mapper Plus (ETM+), and Landsat Operational Land Imager (OLI) data over five years for each city from 2011 to 2015. The Landsat program is one of the most successful missions jointly initiated by U.S. Geological Survey (USGS) and NASA, and it has been serving for the resources mapping, environmental monitoring, and habitability assessment globally since 1972 [42]. In this study, all Landsat 5 TM, Landsat 7 ETM+ scenes, and Landsat 8 OLI scenes are processed through the Level 1 Product Generation System (LPGS). Initially, all images without clouds or with few clouds (less than 30%) were collected from the EarthExplorer platform of USGS, and the cloud-free images over the study sites were refined. Landsat 7 SLC-off ETM+ images were destriped by considering the radiometric characterization of the on-orbit reflective bands [43]. As the stripes are mainly located on the eastern and western edges of each scene due to the scan line corrector failure, the middle area of the scene was selected as the study site to reduce the impacts from the stripes [28]. Atmospheric correction was not applied to the time series Landsat images in this study. Although the multitemporal images were collected in different seasons when the sun-illumination conditions were different, the post-classification change detection analysis strategy was adopted where each image was classified individually with separate training samples. Furthermore, all selected images were cloud free over the study sites. This case was referred to as a classification where the training data and the image were at the same relative scale, and thus, atmospheric correction has little impact on the classification results [44,45,46,47]. Therefore, atmospheric correction is unnecessary in these post-classification change studies [44,48,49]. Subsequently, the images were preprocessed with calibration and geometric correction. Co-registrations were conducted in all images of each city by manually selecting the ground control points (GCPs) with root mean square error (RMSE) values less than one pixel. The co-registration was conducted using the second-order affine transformation implemented in Harris Geospatial’s ENVI software. The temporal distribution of the Landsat images is shown in Figure 3. Each dot represents one image. The day was represented according to the day of year, which is a number ranging from 1 to 365 or 366 in a leap year (e.g., 2012). A total of 93 images were finally selected for the four cities, including 30 images in Melbourne, 22 images in Sao Paulo, 21 images in Hamburg, and 18 images in Chicago. Almost every year had images from each of the four seasons, while some seasons did not have available images due to cloud contaminations, such as the year 2012 in Melbourne, 2011 in Sao Paulo, 2012 in Hamburg, and 2011 and 2013 in Chicago. Especially, for Hamburg and Chicago, there was almost complete snow coverage throughout the winter. Consequently, it was difficult to identify other land cover types from the winter images, and thus only a few winter images were selected for this study. In this study, only one image in 2014 was available in Hamburg, and three images in 2011, 2012, and 2014 were available in Chicago.

## 3. Methods

### 3.1. Reference Data Collection Using Time Series High Resolution Images

Six land cover classes were initially identified, including water bodies, green vegetation, non-photosynthetic vegetation, bright impervious surfaces, dark impervious surfaces, and bare soil. Water bodies include lakes, rivers, ponds, pools, and fountains. Green vegetation includes the land covered by vegetation with green leaves. Green vegetation usually includes tree cover (with canopy cover greater than 10%), shrubberies, grasslands, and fields with crops, while non-photosynthetic vegetation refers to the plants that are unable to carry out photosynthesis processes due to the lack of chloroplasts. The leaves of these plants are often red, orange, brown, or have all fallen off. Impervious surfaces are sites that are dominated by human-built environments, including all non-vegetative, man-made features, such as buildings and roads [50], which rainfall water cannot permeate. In this study, the impervious surface land type is divided into two subtypes: bright impervious surfaces and dark impervious surfaces. Bright impervious surfaces refer to land features that have relatively high reflectance, e.g., cement or concrete roads and bright colored house roofs. Dark impervious surfaces often have relatively lower reflectance, which usually include asphalt roads and dark colored houses. In addition, bare soil is a natural surface with little or no vegetation that is dominated by exposed soil, sand, gravel, or rock backgrounds [51]. The fields without crops are also included. Then, to keep the analysis consistent and compare the four cities, four urban land cover classes were further generated by combining the subclasses of vegetation and impervious surfaces, and finally, impervious surfaces, bare land, vegetation, and water body classes were obtained.

**Figure 4:**
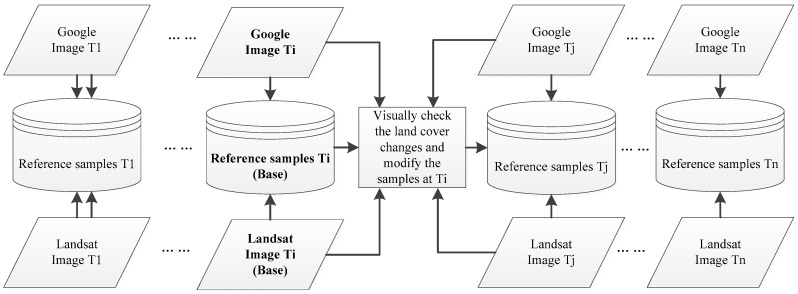
Illustration of the construction of reference samples at time Tj based on the samples at time Ti.

Then, reference samples of each land cover type were collected for each image individually. To keep the samples consistent in different images at different times, a two-step strategy was applied following previous studies [19,51,52,53]. First, a set of samples was collected from the first image under the stratified random sampling scheme [54,55] by visual interpretation over the Landsat image and a corresponding high-resolution image at the nearest time from Google Earth. The visual interpretation over joint Landsat and Google Earth images was assisted with support of ENVI 5.0, which creates KML files of each Landsat images. Afterwards, the KML files were overlaid with the Google Earth images to be visually interpreted. These samples from the first image were treated as the base reference samples. Second, the base samples were checked for if they exhibited any changes in other classes. If there were changes, the sample was removed, or the label was reassigned, and new samples from surrounding sites with more confidence were added [53]. These new samples were treated as the base reference samples for selecting the references samples in the adjacent image. Figure 4 illustrates the generation of reference samples at time Tj based on the reference samples at time Ti. According to similar previous methods of sample collection for time series images, this strategy could reduce the biases that were possibly caused by the sample by using sample pixels that remained the same at different times to train the classifier [19,51,52,53]. Finally, a total of approximately 800 pixels for each land cover class were selected for each image. Subsequently, these samples were empirically divided into training and testing samples with a ratio of approximately 40% vs. 60%.

### 3.2. Land Covers Classification and Accuracy Assessment

In this study, the support vector machine (SVM) was chosen as the classifier. SVM is a binary linear classifier that has advantages regarding small sample identification, noise resistance, and learning efficiency, and it has been applied in many land cover classification studies [56,57,58]. SVM can obtain higher classification accuracy than other traditional methods [59]. SVM uses a kernel function to map non-linear training samples to a high dimensional space so that it can find a linear hyperplane that separates the dataset into a predefined number of classes in this high dimensional space. The radial basis function (RBF) was selected as the kernel function in this study. The one-against-rest strategy was employed for the multiple-class classification. SVM has been widely applied in remote sensing communities, and the success of SVM for urban land cover classification relies on the parameter settings, including the gamma parameter in the RBF and the penalty parameter of a non-linear case in the training samples [59,60,61,62]. In this study, these two parameters were selected with an empirical optimization process that is similar to the cross-validation process [59]. Afterwards, the successful application of SVM in this study depended on the classification scheme and sample design. The next two sections provide details about these two issues.

After all of the images were classified with the training samples, test samples were used to calculate the overall accuracy and kappa coefficient based on the confusion matrix. The accuracies of the classification results were assessed by calculating a confusion matrix, i.e., error matrix. A confusion matrix is calculated by comparing the testing samples and the classification results at corresponding locations [54]. According to the confusion matrix, the overall accuracy can be further derived, which is often used as an indicator to evaluate the classification results. Overall accuracy is calculated by summing the number of pixels that are correctly classified and dividing the sum by the total number of pixels. Moreover, the kappa coefficient measures the overall agreement of the confusion matrix based on the difference between the actual agreement and the chance agreement by calculating the product of the row and column marginals of the confusion matrix [63]. Although the kappa coefficient was criticized for not being robust enough, it has been widely used due to the wide availability in the software packages [16,64]. In this study, we adopted the use of both overall accuracy and the kappa coefficient. The calculations for kappa coefficients can be found in many remote sensing publications [54,55].

### 3.3. Quantification of the Short-Term Change Using Fourier Series and the Sum of Sine Functions

The temporal variations in this study included two categories of variations, short-term land cover changes and long-term land cover changes. Short-term land cover changes may be phenological changes of vegetation and water surfaces (e.g., variable source areas), which could be captured in the images from different seasons. Short-term land cover changes can also be the growth of vegetation (e.g., crops in the farmland) or urban sprawl that is induced by building or pavement construction. Long-term changes can be more complex in diverse ways, such as vegetation succession, afforestation, deforestation, and urbanization. To fit these temporal variations combined with short- and long-term changes, the Fourier series was employed, as it can decompose the complete change into a series of seasonal changes, which may be able to reflect the combinations of different types of changes using their frequencies and amplitudes. A Fourier series is described in Equation (1), where a_0_ models the constant of the change in the temporal variation, *n* is the number of terms in the Fourier series, *w* is the frequency of the related component of the change, *i* is the *i*th term of the series and is related to the frequency of the term, *x* is the time that is an integer representing the acquisition date of each image in this study, and a_0_, *a_i_*, *b_i_*, and *w* are the parameters to be fitted.

(1) $$y=a_{0}+\sum_{i=0}^{n}a_{i}\cos(iwx)+b_{i}\sin(iwx)$$

To investigate the constitutions of these changing amplitudes and frequencies, the fitting coefficients in the Fourier series should be listed and analyzed. In this study, the Fourier series was applied with up to eight different frequencies for fitting. There are 18 coefficients to be fitted by a_0_ to *a*_8_, *b*_1_ to *b*_8_ and the frequency coefficient *w*. Two statistical accuracy measures, R^2^ and root mean square error (RMSE), were employed to assess the fitting results. Additionally, according to the period of sine and cosine function, the period of each item in Equation (1) can be calculated by Equation (2), where π is a constant approximately equal to 3.14159.

(2) $$T=\frac{2\pi }{iw}$$

As a comparison, the Sum of Sine Functions was also employed to quantify the short-term land cover changes. The Sum of Sine Functions is the linear combination of a series of sine functions that can be expressed in Equation (3), which is a periodic function [65]. *a_i_*, *b_i_*, *c_i_*, and *ε* are the parameters to be fitted and *x* is the time that is an integer representing the acquisition date of each image in this study. Therefore, it was employed to fit the periodic changing of different urban land covers in this study. Similar to the Fourier series, the fitting procedure is to estimate the coefficients in the Sum of Sine Functions for each land cover type in the time series change of each city. Empirically, in this study, four components of sine functions were set for the fitting model for Sao Paulo, Hamburg, and Chicago, while seven components were set for the fitting model for Melbourne. That is, there are 13 coefficients to be fitted for Sao Paulo, Hamburg, and Chicago, and 22 coefficients to be fitted for Melbourne.

(3) $$y=\sum_{i=1}^{n}a_{i}\sin(b_{i}x+c_{i})+\varepsilon$$

## 4. Results

### 4.1. Accuracy Assessment of the Multitemporal Satellite Image Classification

**Figure 5:**
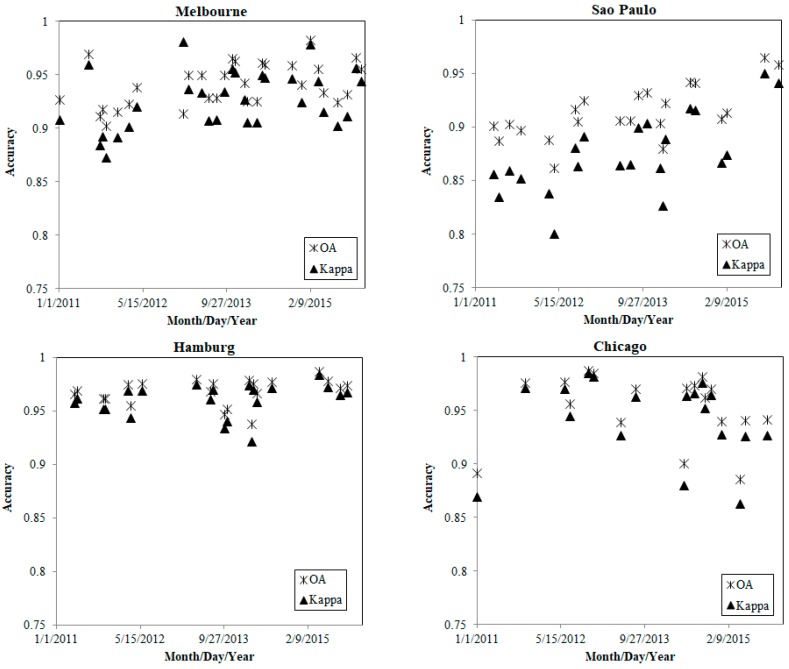
Overall accuracy and kappa coefficient of the urban land cover classification results in the four selected cities; the time scale and accuracy scale are consistent in four cities.

Figure 5 demonstrates the overall accuracy and kappa values for all 94 images over the four cities and the complete time series. First, it was observed that the images were distributed from 2011 to 2015, and the overall accuracy varied from ~86% to ~98% in the different cities, while the kappa values varied from ~0.8 to ~0.98 in the different cities. Generally, Figure 5 indicates that the classification results were reliable with an overall accuracy greater than 86% and a kappa value greater than 0.8. The distribution patterns of the overall accuracy and kappa values were generally consistent, with some slight differences. Second, the accuracy tended to be higher in Hamburg and Chicago than Melbourne and Sao Paulo. Most of the classified images in Hamburg and Chicago reached an overall accuracy of 94% and a kappa coefficient of 0.9. However, most of the classifications in Melbourne and Sao Paulo had overall accuracies of less than 94% and kappa coefficients that were less than 0.92.

### 4.2. The Land Cover Classification and Short-Term Changes

**Figure 6:**
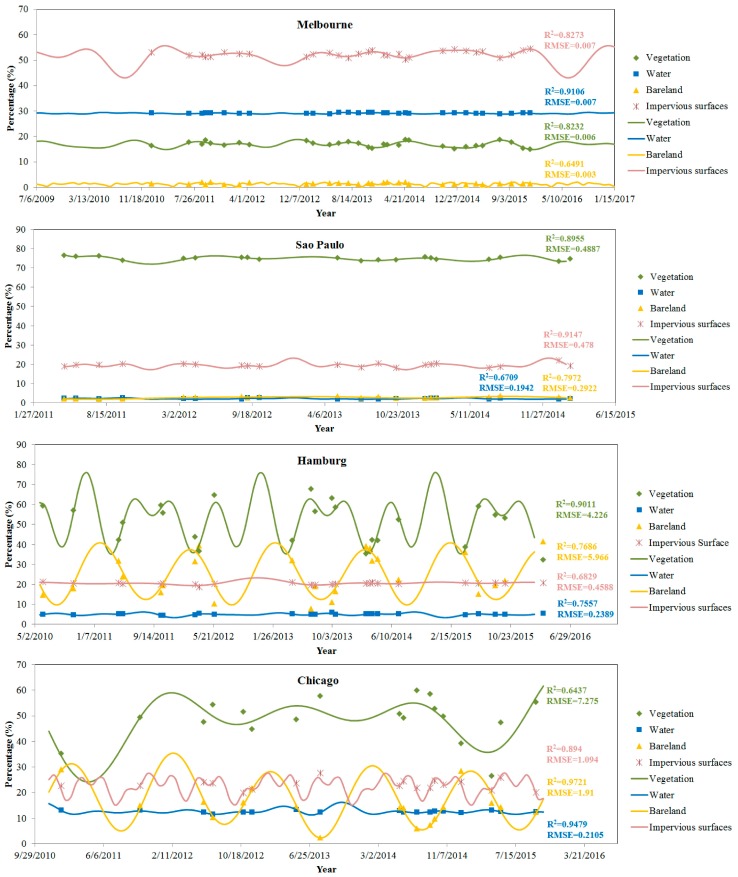
Temporal variations of different urban land cover types fitted using Fourier series.

From the classified land cover images, the area of each land cover type was calculated for each image. Subsequently, the area of each land cover was calculated for each city throughout the time series (Figure 6). The points in Figure 6 are the areas directly calculated from the classified images. Four land cover types, impervious surfaces, water, bare land and vegetation, were represented in different colors. However, it was difficult to determine the changing patterns of the area throughout the time series from only the discrete points.

Several interesting findings can be observed from Figure 6. First, the R-squared value was generally higher than 0.64 for all fittings, and the RMSE was lower than 2%, except for the vegetation and bare land in Hamburg (~4% and 5%, respectively) and vegetation in Chicago (~7%). This result could indicate that the fittings of the Fourier series in all four cities and the different land cover types were good enough to characterize the change features. Second, the landscape patterns are very different in the four selected cities, which can be reflected by the percentages of different land cover types in the four cities. For instance, in Melbourne, impervious surfaces constituted approximately 50% of the study area, water approximately 30%, vegetation approximately 18%, and bare land only approximately 2%. In Sao Paulo, vegetation made up to more than 75% of the study area, while impervious surfaces covered approximately 20% and water and bare land covered approximately 2.5%. Third, the impervious surfaces indicated some fluctuations during the five-year period that appeared to be related to some seasonal factors. This phenomenon is more obvious in Melbourne and Chicago than in the other cities. In particular, the seasonal effect on impervious surfaces in Chicago was more obvious than that in other cities. Although the impervious surfaces were not supposed to exhibit such rises and falls, this phenomenon was also previously reported in Ohio and Indiana, USA [30,31]. The seasonal changes of other land cover types can explain this phenomenon, which can lead to land cover confusions in different seasons. This result can be examined in two aspects: (1) the seasonal changes in precipitation (Figure 2), which directly result in seasonal changes in water surfaces; (2) the seasonal changes in bare land and vegetation are obvious within the five years in Figure 6.

**Figure 7:**
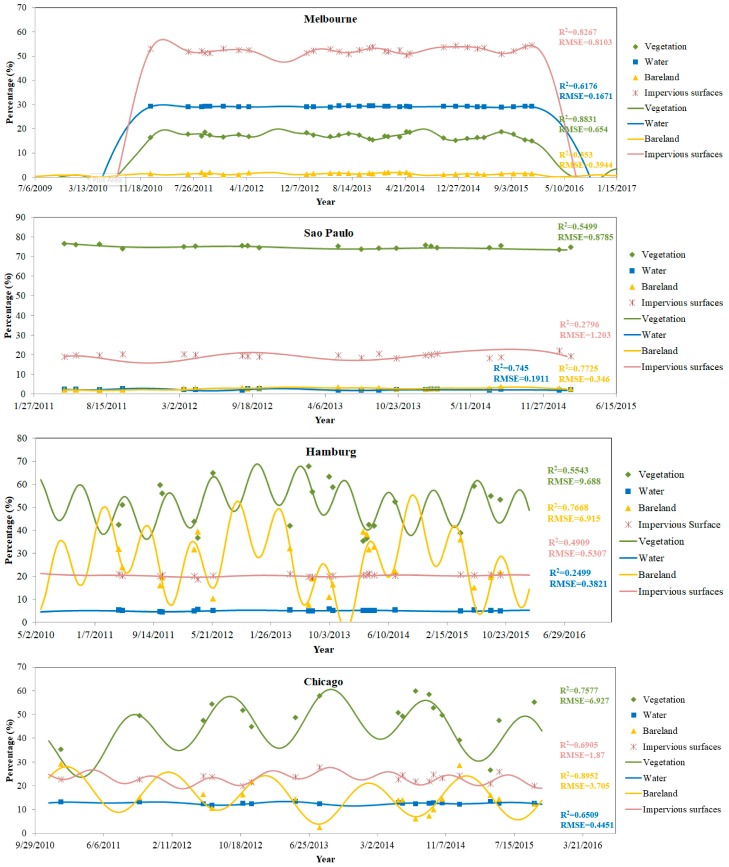
Temporal variations of different urban land cover types fitted using the Sum of Sine Functions.

Comparatively, the Sum of Sine Functions was applied to fit the short-term land cover changes in the four cities. As demonstrated in Figure 7, the fitted patterns are quite different in most of the land covers and cities, especially in Melbourne and Hamburg where negative values were produced. However, in order to make the results more comparable, negative values were not shown by setting the y-axis range to be positive. In comparison with the results in Figure 6, several conclusions can be drawn from Figure 7. First, the R^2^ is generally lower and the RMSE is generally higher, which indicates that the Sum of Sine Functions was not as good as Fourier series for fitting the short-term urban land cover changes. Second, the periods of the Sine functions were not so meaningful. As we calculated the periods, they were mostly several thousand days for most of the land covers changes. Third, negative values occurred in some cases of the fitted results, which were not realistic in the real percentage of land covers. The occurrence of negative values also indicated the low reliability of fitting in some cases, such as in all of the land covers of Melbourne and in the bare land of Hamburg.

### 4.3. Quantification of Short-Term Land Cover Changes

According to the fitting results in Figure 6 and Figure 7, the Fourier series was further used to quantify the short-term land cover changes in different cities. It is noted that the sum of the areas of the four land cover classes was not exactly 100%. One reason is that the modeling of Fourier series was applied to each land cover class individually, without a constraint on the total area of all four land cover classes. Another possible reason is the unclassified pixels during the land cover classification stage. As a result, the sum of four simulated land covers had a variation of less than 10% in most cases, but exceptionally big variations of up to 28% were also observed in some cases, such as the time period of August to December 2012 in Hamburg. However, there were just small number pixels that were unclassified, summing up to less than 1% of the area in each city. Therefore, the major reason should come from the fitting model. Nevertheless, the results illustrate some reasonable short-term changes. For instance, seasonal changes, such as those in vegetation and bare land, were observed with different amplitudes and frequencies of the changes. More specific short-term changes were described, as follows.

Generally, the values of ai and bi indicate the constitutions of amplitudes and frequencies of the final fitted curves (Table 1). The numbers of *a_i_* and *b_i_* indicate the amplitude at the frequency of *iw*. Some major findings can be observed. (1) Melbourne had the highest coefficients of a_0_, indicating that there were no significant seasonal changes; (2) Sao Paulo had the most contribution from a_0_, but the other coefficients also contributed certain amounts to the different land cover classes, such as *b_3_* for impervious surfaces, *b_4_* for bare land, *a_1_*, *b_1_*, and *b_2_* for vegetation, and *a_1_* for water surfaces. This result indicated that vegetation exhibited a greater significant seasonal variation in Sao Paulo. (3) Most of the components contributed to the changes of all four land cover types in Hamburg. However, the contributions of the different components were different. For instance, *a_2_*, *a_3_*, *a_4_* and *a_5_* contributed more to impervious surfaces, while *a_2_* and *b_2_* were significantly more important for bare land. For vegetation, *a_2_*, *b_3_* and *b_4_* made higher contributions than the other components. Changes in the water surfaces were made up of more components from *a_1_* to *b_6_*; and, (4) Chicago experienced significant seasonal variations in all land cover types. In particular, impervious surfaces were observed to undergo seasonal changes at several different frequencies, which can be noted with the coefficients of *a_1_*, *a_2_*, and *b_3_*.

In addition, the period of each item was calculated according to Equation (2). Afterwards, the major or dominant period of each land cover class in each city were calculated according to the largest absolute values of the coefficients (except the constant component a_0_), as shown in Table 2. Some observations can be summarized from Table 2. First, the dominant periods of estimated impervious surfaces were 703.8 days in Melbourne, 702.5 days in Sao Paulo, 687.1 days in Hamburg, and 215.3 days in Chicago. In all cities except Chicago, the changing period for impervious surface was nearly two years. In fact, impervious surfaces in the real world should be constantly growing. However, affected by the limitation of remote sensing observation, the estimated impervious surfaces from satellite images are not exactly those in the real world. This periodic change was caused by the misclassification from confusions with other land cover classes, such as vegetation, bare land and water, which changed seasonally. From Figure 6, it can be observed that the variations in impervious surfaces in Chicago were caused by the significant seasonal changes in vegetation and bare land. These two land cover classes did not consistently change, and thus caused more frequent changes in impervious surfaces. Second, bare land was varied with a period of 99.7 days in Melbourne, 1919.1 days in Sao Paulo, 368.2 days in Hamburg, and 362.5 days in Chicago. In urban areas, the constitutions of bare land may come from bare soil in construction fields or bare soil in vegetated areas. Therefore, bare land can be quite different depending on the urban landscapes of the different cities. Based on the results, the bare land in Hamburg and Chicago mostly changed with a period of one year. The period of bare land change in Melbourne was quite faster and was only one-third of a year, while this period in Sao Paulo was quite long at nearly five years. This result can also be observed in Figure 6, which shows that the bare land in Sao Paulo remained almost consistent within the five-year time series of this study. This result could be caused by two factors: (1) the vegetation cover in Sao Paulo is evergreen and thus does not change to bare soil and (2) the urbanization process was very slow and stable without significant changes within five years. Third, the vegetation changed with a period of 436.6 days in Melbourne, 2211.6 days in Sao Paulo, 183.8 days in Hamburg, and 684.4 days in Chicago. This result can also be observed from Figure 6, which indicates that the evergreen vegetation in Sao Paulo did not change significantly, while the vegetation in Melbourne and Chicago changed gradually not seasonally. Therefore, the periods of vegetation changes in these two cities were from 1.5 to two years. In Hamburg, the vegetation changed faster than in the other cities, as shown in Figure 6, which is consistent with the 183.8 days in Table 2. Finally, water surfaces continuously changed with a period of 384.9 days in Melbourne, 469.6 days in Sao Paulo, 285.0 days in Hamburg, and 181.6 days in Chicago. When compared to bare land and vegetation, water surfaces were not affected by many factors, and thus they remained more consistent with the normal climate period throughout a year. Therefore, the periods in these four cities varied from one-half of a year to one and half years, which was the most stable among the land cover classes.

## 5. Discussion

### 5.1. How The Methods and Outcome Could Be Applicable in Urban Monitoring and Planning

This study develops a new strategy for reference samples collection for the time series classification of urban land covers. The proposed strategy incorporates both the Landsat satellite images and high resolution Google Earth images in terms of time series changes. The results proved the effectiveness of the proposed strategy with high accuracy of time series land cover classification. The sampling strategy is applicable in various studies related to the processing of time series satellite images, such as time series analysis or long-term change detection of land surfaces in urban, agriculture, environment, and ecological systems. In urban monitoring, this sampling strategy can be applied to various scales from local city, regional metropolitans, and national and global urbanization monitoring using free of charge satellite datasets, such as Landsat and MODIS images.

The quantification of short-term urban land cover changes derived from the fitting of periodic functions provides scientific support to decision makers of urban planning and management. First, for regular land use and land cover survey of a city, it is often conducted for a time interval of one year, three years, or five years, which are defined as long-term in this study. Subsequently, one question is which season should be the best to conduct the survey of a city. The outcome of this study indicates that different cities may have different characteristics due to the short-term land cover changes, and this short-term change varies from city to city depending on the land cover types, climates, and urban landscapes. Therefore, the decision makers should carefully examine these factors before they make a plan for the regular land use and land cover survey of their cities. Second, this study makes a recommendation of Fourier series as a tool to analyze the short-term land cover changes to urban planners to quantitatively understand the short-term changing patterns of local cities. When compared with the Sum of Sine Functions, Fourier series come out to provide more reliable and more stable results. Third, the study highlights the use of free of charge satellite images in urban monitoring and planning. Actually, local urban planners often employ high resolution remote sensing images for geospatial planning using high resolution commercial satellites or airborne or Unmanned Aerial Vehicle (UAV) sensors, which are often expensive, especially when dealing with regional cities or metropolitan. The outcome of this study demonstrates the benefits of public medium resolution satellite images to understand the short-term changing patterns in urban land covers. These satellite images are freely accessible with high temporal resolutions from one day to half month from coarse to medium spatial resolutions. They can provide quantification of short-term land cover changes to decision makers as their scientific reference of long-term planning for urban land use and land cover survey.

### 5.2. Limitations of the Methodology

Firstly, one limitation of the study is the selection of study sites. Due to the limited computing capability, only four cities were selected for comparing the short-term land covers dynamics. The selected cities are from north and south spheres, from different climate zones, and also from developed and developing countries. However, there are lots of cities in the world with various climate types, topography, landscapes, and urban development policies. Including more cities would certainly be better to improve the confidence of study results and conclusions. To overcome the computational limits, clouding computing platforms, such as Google Earth Engine, could provide good opportunity for including many more cities for such studies.

Secondly, as mentioned in Section 4.3 about the quantification of the short-term land cover changes, the total percentage of four land covers types from the fitted Fourier series model or Sum of Sine Functions were not exactly 100%. The major reason came from the fitting model, which was not forced to 100% for the sum of four land covers. However, according to our knowledge, in order to investigate the periodic changes of different land covers, we need to fit them individually; otherwise the periods would not be successful with Fourier series. From the results, the fitting models from the Fourier series or Sum of Sine Functions were still sensible as each land cover was analyzed individually, as explained in Section 4.3. Nevertheless, we found that the unconstrained Fourier series model can produce significant errors in some cases. For instance, during the period of August to December 2012 in Hamburg, there was a difference of up to 28% from 100%. In this case, the fitted results were not reliable enough by using this model. Therefore, the unconstrained Fourier series and Sum of Sine Functions should be improved by adding the constraint of 100%. However, the technical difficulty remains unsolved in this study. Moreover, the study was also limited by the lack of datasets in winter seasons in Hamburg and Chicago where snow covers the urban surfaces throughout the winter. Future studies should be conducted to improve the fitting of modeling the periodic changes of different land covers.

Thirdly, time scale is one of the most important factors necessary to quantify short-term land cover changes. Some of the changes are seasonal or occur within even shorter periods. According to the results in Section 4.2, the estimated period ranges from tens of days to hundreds of days. However, this result was affected by the time scale of the available satellite data. Although the revisit time of Landsat is 16 days, the Landsat images were not available in specific cities every 16 days due to factors, such as clouds contamination. In fact, images in every month or every season are often difficult to obtain. Consequently, some seasonal changes cannot be properly observed from these satellite images, which may result in the longer periods in this study. To overcome this limitation, satellites with higher temporal resolution or multisource satellite data fusion could provide a good solution. For instance, with the increasing availability of Sentinel 2 data, the temporal resolution can be improved to five days, which provides good opportunities for obtaining more images at a shorter timescale. Moreover, multisource data fusion between Landsat and MODIS data is also an important technology that can be utilized to enhance such studies.

### 5.3. Uncertainty of the Fourier Series Fitting

Several factors could result in the uncertainty of the quantified short-term land cover change that is illustrated and discussed in Section 4.2. These factors include both the classification of time series images and the fitting of land cover changes using the Fourier series. Therefore, Table 3 summarizes the classification accuracy, fitting errors, the number of parameters in the Fourier series and the number of observations, i.e., the number of time series images in each city and each land cover type. First, the classification accuracy results were quite good in all four cities with an overall accuracy of greater than 86% and a kappa coefficient greater than 0.8001. In particular, most of the classifications reached accuracies higher than 90%. Second, most of the fittings had R-squared values higher than 0.8 and RMSE values lower than 0.5% for each land cover area. In terms of the R-squared, the fittings of bare land in Melbourne, water surfaces in Sao Paulo, impervious surfaces in Hamburg, and vegetation in Chicago were not as confident with R-squared values that were lower than 0.7. In terms of the RMSE, the bare land and vegetation in Hamburg and vegetation in Chicago were fitted with moderate RMSE values that were greater than 4%. Therefore, when considering the data availability, the fitting results of Melbourne and Sao Paulo are more reliable than Chicago and Hamburg. Third, for the parameter estimations from the Fourier series, the number of observations was generally 2–3 times greater than the number of parameters. Theoretically, the estimated parameters were not perfect due to the limited number of images available in each city. In particular, for some land cover classes in some cities (Table 3), the number of parameters was slightly less than two times the number of observations, and thus this condition reduced the confidence of the fitting results. However, the fitting errors and R-squared values indicated that the land cover classes (e.g., bare land in Hamburg) with fewer parameters and more observations did not necessarily reach low RMSE and high R-squared values, which indicated that a comprehensive design and consideration should be adopted to reduce the uncertainty and increase the confidence of the quantification of short-term land cover changes.

### 5.4. Recommendations for Future Studies

When considering the uncertainty of the Fourier series fitting and the availability limitations of the satellite images, one future direction is to reduce the uncertainty by improving the fitting methods and increasing the satellite data availability by combining multiple data sources. In addition, we emphasize that the classification accuracy fluctuates along different short-term periods, as observed in this study. Short-term and long-term multitemporal or time series satellite data have been intensively used in many applications, such as the land use or land cover changes using the post-classification analysis strategy. However, the fluctuations or changing patterns of classification accuracy have seldom been reported, which may cause some uncertainty that is related to the results. The changes in classification accuracy can be caused by different factors, including seasonal changes in vegetation, water and bare soil, the selection of study areas, the selection of satellite data, and the classification methods. While considering these factors, it is recommended that future long-term studies of land use or land cover change analysis using satellite images select appropriate seasons when the classification accuracy is higher. However, a general pattern of the accuracy changes cannot be derived from this study due to the availability of satellite images and the limitation of the number of cities. Therefore, a short-term pattern analysis of land cover classification accuracy change is recommended to be examined for better data selection in long-term studies.

## 6. Conclusions

This study proposed a methodology to quantify short-term urban land cover changes using time series satellite images. The methodology mainly includes a strategy of collecting time series reference samples jointly from Landsat images and Google Earth images, and a change modelling procedure by fitting a periodic function. Short term changes in urban areas are important phenomenon, as it is related to rapid urbanization, seasonal climate changes, and phenological changes of urban ecosystems. There is lack of understanding to these short-term characteristics in existing literature because of the lack of time series data sets. A better understand of these short term changes is beneficial in various aspects in urban planning and management. This study quantified the short-term urban land cover changes of four cities using time series Landsat images within a period between 2011 and 2015 with a comparison study in Melbourne, Sao Paulo, Hamburg and Chicago. The Fourier series was employed to model the temporal variations of different land cover classes, with a R-squared value higher than 0.80 and a root mean square error lower than 7.2% of the area in each city. Several conclusions were drawn from the experiments: (1) bare land and vegetation were the most varying land cover classes with periods from less than one year to more than five years, as bare land and vegetation consisted of different subtypes and were inter-changing at different times. Bare land and vegetation in Sao Paulo changed very slowly with a period of approximately five years; (2) water surface was the most stable land cover class among the land cover classes, since it was not affected by many factors. The changing periods were generally from half a year to one and a half years in different areas; and, (3) urban impervious surfaces changed with a period of approximately 700 days in Melbourne, Sao Paulo, and Hamburg, and a period of approximately 215 days in Chicago, and these changes were actually caused by the unavoidable misclassification from confusions between impervious surfaces and vegetation, bare land, and water due to their spectral similarities and overlay relationships in urban landscapes. These seasonal characteristics provided important information for the monitoring and assessment of urban areas using satellite remote sensing technology.

## Figures and Tables

**Table 1:** Fitting coefficients of the Fourier series for the different land cover (LC) types in four cities; Melbourne is presented as M, Sao Paulo as S, Hamburg as H, and Chicago as C. Impervious surfaces are abbreviated as IS, bare land as BAR, vegetation as VEG, and water as WAT.

	LC	a_0_	*a*_1_	*b*_1_	*a*_2_	*b*_2_	*a*_3_	*b*_3_	*a*_4_	*b*_4_	*a*_5_	*b*_5_	*a*_6_	*b*_6_	*a*_7_	*b*_7_	*a*_8_	*b*_8_
**M**	IS	51.67	0.208	−0.787	−0.468	−1.304	1.442	−0.916	0.3789	0.302	0.4608	1.474	−1.434	1.26	−0.466	−0.165	−0.577	−0.693
	BAR	1.355	−0.175	0.3015	0.0962	0.1316	0.147	−0.124	0.1591	0.0537	0.02	0.0664	−0.00704	−0.169	−0.063	−0.00023	−0.232	−0.089
	VEG	16.91	−0.256	−0.231	−0.095	0.053	0.0927	−0.152	−0.627	−0.557	0.2491	−0.58	0.0499	−0.257	−0.228	0.7164		
	WAT	29.12	0.087	0.0144	0.0962	−0.059	0.0978	0.0372	−0.076	−0.069	−0.017	−0.00536	−0.00594	0.00182	−0.053	0.0492	0.0322	0.0981
**S**	IS	19.52	0.2213	−0.7699	−0.6136	−0.4874	0.3388	0.9038	0.0926	0.4768	0.001217	−0.6547	−0.4272	−0.5066				
	BAR	2.627	−0.2989	−0.3105	0.1152	−0.1482	−0.2444	0.1336	−0.125	0.2058								
	VEG	186.1	70.75	194.7	−127.4	107.1	−98.11	−56.41	9.845	−63.34	27.9	−5.96	4.201	6.856				
	WAT	2.167	0.1443	−0.2204	0.009498	−0.04595	0.03229	−0.09973	−0.04569	−0.09476								
**H**	IS	20.72	0.1891	0.1291	0.5941	0.02974	0.6129	0.05458	0.5256	0.06834	0.4948	−0.1312	0.1416	0.1216				
	BAR	25.09	1.122	1.313	4.176	13.37	0.9052	0.3479										
	VEG	53.04	−0.2223	2.937	6.231	1.791	−4.674	5.594	−8.729	7.167								
	WAT	4.939	−0.2095	0.0559	−0.2429	0.1876	0.2851	0.2282	0.4525	−0.1428	0.2119	−0.05687	−0.2976	−0.2312				
**C**	IS	22.6	1.402	0.6598	1.603	−2.814	−0.3411	1.276	0.012	0.8081	−2.761	0.2241						
	BAR	18.03	−1.773	−0.4405	0.5072	1.657	1.701	−0.1048	−0.5685	0.4593	12.59	−1.947						
	VEG	48.67	−0.3125	2.776	1.664	4.721	8.529	−5.033	−6.367	−5.891								
	WAT	12.87	0.551	0.3425	0.2042	0.3781	−0.3731	−0.1885	−0.6173	−0.5841	−0.03062	−0.6431	0.3435	−0.7094				

Note: Melbourne was presented as M, Sao Paulo as S, Hamburg as H, and Chicago as C. Impervious surface was abbreviated as IS, bare land as BAR, vegetation as VEG, and water as WAT.

**Table 2:** The dominant period of each fitted urban land cover variation curves.

City	Land Cover	Period (Days)	City	Land Cover	Period (Days)
Melbourne	IS	703.8	Hamburg	IS	687.1
	BAR	99.7		BAR	368.2
	VEG	436.6		VEG	183.8
	WAT	384.9		WAT	285.0
Sao Paulo	IS	702.5	Chicago	IS	215.3
	BAR	1919.1		BAR	362.5
	VEG	2211.6		VEG	684.4
	WAT	469.6		WAT	181.6

**Table 3:** Factors of uncertainty in the Fourier series fitting.

City	Land Cover	Number of Parameters	Number of Observations	R-Squared	RMSE (%)	Overall Accuracy (%)	Kappa Coefficient
**M**	IS	17	30	0.8273	0.007	90.20~98.24	0.8723~0.9806
	BAR	17	30	0.6491	0.003		
	VEG	15	30	0.8232	0.006		
	WAT	17	30	0.9106	0.007		
**S**	IS	13	22	0.9147	0.4780	86.19~96.49	0.8001~0.9497
	BAR	9	22	0.7972	0.2922		
	VEG	13	22	0.8955	0.4887		
	WAT	9	22	0.6709	0.1942		
**H**	IS	13	24	0.6829	0.4588	93.74~98.68	0.9217~0.9835
	BAR	7	24	0.7686	5.966		
	VEG	9	24	0.9011	4.226		
	WAT	13	24	0.7557	0.2389		
**C**	IS	11	18	0.8940	1.094	88.56~98.72	0.9847~0.8627
	BAR	11	18	0.9721	1.910		
	VEG	9	18	0.6437	7.275		
	WAT	13	18	0.9479	0.2105		

## References

[1] Puertas O.L., Brenning A., Meza F.J. (2013). Balancing misclassification errors of land cover classification maps using support vector machines and Landsat imagery in the Maipo river basin (central chile, 1975–2010). Remote Sens. Environ..

[2] Seto K.C., Guneralp B., Hutyra L.R. (2012). Global forecasts of urban expansion to 2030 and direct impacts on biodiversity and carbon pools. Proc. Natl. Acad. Sci. USA.

[3] Lambin E.F., Turner B.L., Geist H.J., Agbola S.B., Angelsen A., Bruce J.W., Coomes O.T., Dirzo R., Fischer G., Folke C. (2001). The causes of land-use and land-cover change: Moving beyond the myths. Glob. Environ. Chang..

[4] Weng Q.H. (2012). Remote sensing of impervious surfaces in the urban areas: Requirements, methods, and trends. Remote Sens. Environ..

[5] Small C. (2005). A global analysis of urban reflectance. Int. J. Remote Sens..

[6] Poursanidis D., Chrysoulakis N., Mitraka Z. (2015). Landsat 8 vs. Landsat 5: A comparison based on urban and pen-urban land cover mapping. Int. J. Appl. Earth Obs. Geoinf..

[7] Collins J.B., Woodcock C.E. (1996). An assessment of several linear change detection techniques for mapping forest mortality using multitemporal Landsat TM data. Remote Sens. Environ..

[8] Rokni K., Ahmad A., Selamat A., Hazini S. (2014). Water feature extraction and change detection using multitemporal Landsat imagery. Remote Sens..

[9] Alonso A., Munoz-Carpena R., Kennedy R.E., Murcia C. (2016). Wetland landscape spatio-temporal degradation dynamics using the new Google Earth Engine cloud-based platform: Opportunities for non-specialists in remote sensing. Trans. ASABE.

[10] Munyati C. (2000). Wetland change detection on the Kafue Flats, Zambia, by classification of a multitemporal remote sensing image dataset. Int. J. Remote Sens..

[11] Hui F.M., Xu B., Huang H.B., Yu Q., Gong P. (2008). Modelling spatial-temporal change of Poyang lake using multitemporal Landsat imagery. Int. J. Remote Sens..

[12] Shelestov A., Lavreniuk M., Kussul N., Novikov A., Skakun S. (2017). Exploring Google earth engine platform for Big Data Processing: Classification of multi-temporal satellite imagery for crop mapping. Front. Earth Sci..

[13] Dong J.W., Xiao X.M., Menarguez M.A., Zhang G.L., Qin Y.W., Thau D., Biradar C., Moore B. (2016). Mapping paddy rice planting area in northeastern Asia with Landsat 8 images, phenology-based algorithm and Google Earth Engine. Remote Sens. Environ..

[14] Roodposhti M.S., Aryal J., Bryan B.A. (2018). A novel algorithm for calculating transition potential in cellular automata models of land-use/cover change. Environ. Modell. Softw..

[15] Anees A., Aryal J., O’Reilly M.M., Gale T.J. (2016). A relative density ratio-based framework for detection of land cover changes in MODIS NDVI time series. IEEE J. Sel. Top. Appl. Earth Obs. Remote Sens..

[16] Stein A., Aryal J., Gort G. (2005). Use of the Bradley-Terry model to quantify association in remotely sensed images. IEEE Trans. Geosci. Remote Sens..

[17] Bruzzone L., Prieto D.F. (2002). An adaptive semiparametric and context-based approach to unsupervised change detection in multitemporal remote-sensing images. IEEE Trans. Image Process..

[18] Byrne G.F., Crapper P.F., Mayo K.K. (1980). Monitoring land-cover change by principal component analysis of multitemporal Landsat data. Remote Sens. Environ..

[19] Li X., Gong P., Liang L. (2015). A 30-year (1984–2013) record of annual urban dynamics of Beijing city derived from Landsat data. Remote Sens. Environ..

[20] Song K.Y., Zhao J.Y., Ouyang W., Zhang X., Hao F.H. (2010). LUCC and landscape pattern variation of wetlands in warm-rainy Southern China over two decades. Procedia Environ. Sci..

[21] Seto K.C., Woodcock C.E., Song C., Huang X., Lu J., Kaufmann R.K. (2002). Monitoring land-use change in the Pearl River Delta using Landsat TM. Int. J. Remote Sens..

[22] Tsutsumida N., Comber A.J. (2015). Measures of spatio-temporal accuracy for time series land cover data. Int. J. Appl. Earth Obs. Geoinf..

[23] Jansen L.J.M., Bagnoli M., Focacci M. (2008). Analysis of land-cover/use change dynamics in Manica Province in Mozambique in a period of transition (1990–2004). For. Ecol Manag..

[24] Powell S.L., Cohen W.B., Yang Z., Pierce J.D., Alberti M. (2008). Quantification of impervious surface in the Snohomish water resources inventory area of western Washington from 1972–2006. Remote Sens. Environ..

[25] Suarez-Rubio M., Lookingbill T.R., Elmore A.J. (2012). Exurban development derived from Landsat from 1986 to 2009 surrounding the district of Columbia, USA. Remote Sens. Environ..

[26] Sexton J.O., Song X.-P., Huang C., Channan S., Baker M.E., Townshend J.R. (2013). Urban growth of the Washington, DC–Baltimore, MD metropolitan region from 1984 to 2010 by annual, Landsat-based estimates of impervious cover. Remote Sens. Environ..

[27] Song X.-P., Sexton J.O., Huang C., Channan S., Townshend J.R. (2016). Characterizing the magnitude, timing and duration of urban growth from time series of Landsat-based estimates of impervious cover. Remote Sens. Environ..

[28] Zhang H.S., Zhang Y.Z., Lin H. (2014). Seasonal effects of impervious surface estimation in subtropical monsoon regions. Int. J. Digit. Earth.

[29] Zhang L., Weng Q.H. (2016). Annual dynamics of impervious surface in the Pearl River Delta, China, from 1988 to 2013, using time series Landsat imagery. ISPRS J. Photogramm. Remote Sens..

[30] Weng Q.H., Hu X.F., Liu H. (2009). Estimating impervious surfaces using linear spectral mixture analysis with multitemporal ASTER images. Int. J. Remote Sens..

[31] Wu C.S., Yuan F. (2007). Seasonal sensitivity analysis of impervious surface estimation with satellite imagery. Photogramm. Eng. Remote Sens..

[32] Xu R., Zhang H., Lin H. (2018). Annual dynamics of impervious surfaces at city level of Pearl River Delta metropolitan. Int. J. Remote Sens..

[33] Walker J.J., de Beurs K.M., Henebry G.M. (2015). Land surface phenology along urban to rural gradients in the US Great Plains. Remote Sens. Environ..

[34] Zhang X.Y., Friedl M.A., Schaaf C.B., Strahler A.H., Schneider A. (2004). The footprint of urban climates on vegetation phenology. Geophys. Res. Lett..

[35] Xie Y.C., Fan S.Y. (2014). Multi-city sustainable regional urban growth simulation-MSRUGS: A case study along the mid-section of Silk Road of China. Stoch. Environ. Res. Risk Assess..

[36] Romolini M., Grove J.M., Locke D.H. (2013). Assessing and comparing relationships between urban environmental stewardship networks and land cover in Baltimore and Seattle. Landsc. Urban Plan..

[37] Yuan F., Sawaya K.E., Loeffelholz B.C., Bauer M.E. (2005). Land cover classification and change analysis of the Twin Cities (Minnesota) Metropolitan Area by multitemporal Landsat remote sensing. Remote Sens. Environ..

[38] Bauer M.E., Yuan F., Sawaya K.E. (2004). Multi-temporal Landsat image classification and change analysis of land cover in the Twin Cities (Minnesota) Metropolitan area. Analysis of Multi-Temporal Remote Sensing Images.

[39] Kottek M., Grieser J., Beck C., Rudolf B., Rubel F. (2006). World map of the Köppen-Geiger climate classification updated. Meteorol. Musikz..

[40] Van Leeuwen C.J. (2017). Water governance and the quality of water services in the city of Melbourne. Urban Water J..

[41] Notteboom T.E. (2010). Concentration and the formation of multi-port gateway regions in the European container port system: An update. J. Transp. Geogr..

[42] Lauer D.T., Morain S.A., Salomonson V.V. (1997). The Landsat program: Its origins, evolution, and impacts. Photogramm. Eng. Remote Sens..

[43] Scaramuzza P.L., Markham B.L., Barsi J.A., Kaita E. (2004). Landsat-7 ETM+ on-orbit reflective-band radiometric characterization. IEEE Trans. Geosci. Remote Sens..

[44] Song C., Woodcock C.E., Seto K.C., Lenney M.P., Macomber S.A. (2001). Classification and change detection using Landsat TM data: When and how to correct atmospheric effects?. Remote Sens. Environ..

[45] Kawata Y., Ohtani A., Kusaka T., Ueno S. (1990). Classification accuracy for the MOS-1 MESSR data before and after the atmospheric correction. IEEE Trans. Geosci. Remote Sens..

[46] Forster B.C. (1984). Derivation of atmospheric correction procedures for Landsat MSS with particular reference to urban data. Int. J. Remote Sens..

[47] Fraser R.S., Bahethi O.P., Al-Abbas A.H. (1977). The effect of the atmosphere on the classification of satellite observations to identify surface features. Remote Sens. Environ..

[48] Foody G.M., Palubinskas G., Lucas R.M., Curran P.J., Honzak M. (1996). Identifying terrestrial carbon sinks: Classification of successional stages in regenerating tropical forest from Landsat TM data. Remote Sens. Environ..

[49] Singh A. (1989). Digital change detection techniques using remotely-sensed data. Int. J. Remote Sens..

[50] Schneider A., Mertes C.M. (2014). Expansion and growth in Chinese cities, 1978–2010. Environ. Res. Lett..

[51] Gong P., Wang J., Yu L., Zhao Y.C., Zhao Y.Y., Liang L., Niu Z.G., Huang X.M., Fu H.H., Liu S. (2013). Finer resolution observation and monitoring of global land cover: First mapping results with Landsat TM and ETM+ data. Int. J. Remote Sens..

[52] Schneider A. (2012). Monitoring land cover change in urban and pen-urban areas using dense time stacks of Landsat satellite data and a data mining approach. Remote Sens. Environ..

[53] Grinand C., Rakotomalala F., Gond V., Vaudry R., Bernoux M., Vieilledent G. (2013). Estimating deforestation in tropical humid and dry forests in Madagascar from 2000 to 2010 using multi-date Landsat satellite images and the random forests classifier. Remote Sens. Environ..

[54] Jensen J.R. (2007). Introductory Digital Image Processing: A Remote Sensing Perspective.

[55] Zhang H.S., Lin H., Li Y., Zhang Y.Z. (2013). Feature extraction for high-resolution imagery based on human visual perception. Int. J. Remote Sens..

[56] Vapnik V. (1995). The Nature of Statistical Learning Theory.

[57] Vapnik V. (1998). Statistical Learning Theory.

[58] Kolios S., Stylios C.D. (2013). Identification of land cover/land use changes in the greater area of the Preveza peninsula in Greece using Landsat satellite data. Appl. Geogr..

[59] Zhang H.S., Zhang Y.Z., Lin H. (2012). A comparison study of impervious surfaces estimation using optical and SAR remote sensing images. Int. J. Appl. Earth Obs. Geoinf..

[60] Sukawattanavijit C., Chen J., Zhang H.S. (2017). GA-SVM algorithm for improving land-cover classification using SAR and optical remote sensing data. IEEE Trans. Geosci. Remote Sens..

[61] Zhang Y.Z., Zhang H.S., Lin H. (2014). Improving the impervious surface estimation with combined use of optical and SAR remote sensing images. Remote Sens. Environ..

[62] Mountrakis G., Im J., Ogole C. (2011). Support vector machines in remote sensing: A review. ISPRS J. Photogramm. Remote Sens..

[63] Congalton R.G., Oderwald R.G., Mead R.A. (1983). Assessing Landsat classification accuracy using discrete multivariate-analysis statistical techniques. Photogramm. Eng. Remote Sens..

[64] Pontius R.G., Millones M. (2011). Death to kappa: Birth of quantity disagreement and allocation disagreement for accuracy assessment. Int. J. Remote Sens..

[65] Fan S.K.S., Chang Y.J., Aidara N. (2013). Nonlinear profile monitoring of reflow process data based on the sum of sine functions. Qual. Reliab. Eng. Int..

